# Predicting Changes in Systolic and Diastolic Blood Pressure of Hypertensive Patients in Indonesia Using Machine Learning

**DOI:** 10.1007/s11906-023-01261-5

**Published:** 2023-08-29

**Authors:** Desy Nuryunarsih, Lucky Herawati, Atik Badi’ah, Jenita Doli Tine Donsu, Okatiranti

**Affiliations:** 1 School of Health and Life Sciences, Glasgow Caledonian University, Cowcaddens Rd, Glasgow, G4 0BA UK; 2 Environmental Health Department, Health Polytechnic, Ministry of Health, Yogyakarta, Indonesia; 3 Nursing Department, Health Polytechnic, Ministry of Health, Yogyakarta, Indonesia; 4 Nursing Department, University of Nottingham, Nottingham, UK

**Keywords:** Machine learning, Hypertension, Systolic and diastolic pressure, Risk factors

## Abstract

**Purpose of Review:**

This retrospective study investigated factors that influence the occurrence of decreased systolic and diastolic blood pressure including sociodemographic and economic factors, hypertension duration, cigarette consumption, alcohol consumption, duration of smoking, type of cigarettes, exercise, salt consumption, sleeping pills consumption, insomnia, and diabetes. These factors were applied to predict the reality of systolic and diastolic decrease using the machine learning algorithm Naïve Bayes, artificial neural network, logistic regression, and decision tree.

**Recent Findings:**

The increase in blood pressure, both systolic and diastolic, is very harmful to the health because uncontrolled high systolic and diastolic blood pressure can cause various diseases such as congestive heart failure, kidney failure, and cardiovascular disease. There have been many studies examining the factors that influence the occurrence of hypertension, but few studies have used machine learning to predict hypertension.

**Summary:**

The machine learning models performed well and can be used for predicting whether a person with hypertension with certain characteristics will experience a decrease in their systolic or diastolic blood pressure after treatment with antihypertensive drugs.

## Introduction

The increase in blood pressure both systolic and diastolic is very harmful to the health because high blood pressure can cause various diseases such as congestive heart failure, kidney failure, and cardiovascular disease [1–4]. Systolic blood pressure (SBP) is generated when the heart pumps blood throughout the body, while diastolic blood pressure (DBP) occurs when the heart is resting and filled with blood [5]. If only SBP increases while diastolic remains, it is still very dangerous for the individual [6]. Isolated systolic hypertension (ISH) is defined as SBP ≥ 140 mmHg and DBP < 90 mmHg [7]. Several studies showed that isolated systemic hypertension, which is most common in people over 60 years old, can be caused by arterial stiffness, an overactive thyroid (hyperthyroidism), diabetes, heart valve disease, and obesity [7–11]. Furthermore, uncontrolled ISH can eventually lead to an increased risk of stroke, myocardial infarction, heart failure, peripheral vascular disease, aneurysm, chronic kidney disease, retinopathy, and erectile dysfunction [12, 13]. Similarly, isolated diastolic hypertension (IDH) (systolic < 160 mmHg and diastolic > 90 mmHg) [14] is often associated with an increased risk of a disease of the aorta [15]. The aorta carries blood and oxygen to the heart; therefore, people with increased diastolic pressure are usually more susceptible to abdominal aortic aneurysm, which, if ruptures, can cause death [16].

Thus, lowering SBP and DBP is very important. This can be achieved by antihypertensive drugs, lifestyle modifications such as aerobic exercise, low salt (sodium) diet, and maintaining a healthy weight balance. Excessive weight, especially in the abdomen, can cause an increase in blood pressure between 18.5 to 24.9 kg/m^2^, and if a person is obese, then weight loss becomes important, alcohol consumption should be limited, and smoking should be stopped immediately as it can cause plaque buildup in the artery walls and leads to high blood pressure [17, 18]. Adequate sleep is also very important and sleep experts recommend sleeping as much as 7 to 8 h every night as sleeping less than 6 h a day may increase blood pressure and worsen the hypertensive condition [19].

Machine learning (ML) is a well-known artificial intelligence (AI) technology that is developing very rapidly and is now the most popular in the fourth industrial revolution (industry 4.0). Applying machine learning to many tasks that usually require several stages of calculations using statistics can be achieved more rapidly [20, 21]. Islam et al. predicted hypertension incidence using several risk factors as predictors and several machines learning methods including decision tree (DT), random forest (RF), gradient boosting machine (GBM), extreme gradient boosting (XGBoost), linear discriminant analysis (LDA), and logistic regression (LoR) [22••]. Our study applied several machine learning predictive models to data randomly selected from the medical records of several community health centers in Indonesia to predict a decrease in diastolic and systolic pressure status in hypertensive patients with a smoking history.

## Method

This retrospective study was conducted to develop a model to predict whether a patient who has a history of smoking has decreased systolic and diastolic pressure after taking medicines. The study involved 100 hypertension male patients from the Gamping 1 and Gamping 2 Health Center, Sleman Regency, Yogyakarta, who had a smoking history. This study was approved by the Health Research Ethics Commission of the Yogyakarta Ministry of Health (No. e-KEPK/POLKESYO/0646.1/X/2022).

The datasets were classified or grouped using machine learning into input and output features. The input features (*X*) are explained further in detail later and the output variables (*y*1) were a decrease in SBP and (*y*2) for DBP. The output variable, called the target feature, was binomial data. The model prediction algorithms used were NB (Naïve Bayes), ANN (artificial neural network), LoR (logistic regression), and DT (decision tree).

The logistic regression model was trained using observation features and their respective labels and then used to predict new data. Binary classification predictions, i.e., generating yes or no answers were used, which is another form of linear regression which uses binary instead of numeric categories [21].

NB is a set of classification algorithms built on the theory of Bayes [23] that calculate how much the high probability of an example in an observation entering a specific group (class). The Bayes method classification model utilizes a training dataset to calculate each class’s probability based on its feature values. When the model is confronted with new data, new features are used to calculate which classes will likely be high. In statistics, this theory explains the so-called conditional probability [21], which is the possibility of event A occurring if event B appears, and since event A depends on event B, this is conditional.

A decision tree is a machine learning algorithm that uses a set of rules to make decisions with a tree-like structure that models possible outcomes. By breaking down data into smaller groups based on the data attributes, the division of these groups is repeated so that all data elements belonging to the same class fit into one group. This is similar to the way humans think differently; humans think based on experience, while in DT, computers break down data by measuring information gain or input information in the form of features [21].

ANN are algorithms that use the principle of probability to create a classification prediction model and by utilizing data on past events, the model can predict what will happen in the future. This model calculates the probability of an event and can change if additional supporting information is provided [21]. ANN has a black box approaching method, meaning that what is happening inside the process cannot be seen clearly from the outside. The model is formed by complex mathematical calculus that is difficult to understand, but this does not prevent the implementation of neural networks in many scientific practices because of their ability to capture the operation characteristics with a good degree of accuracy [24].

The performance of the models was evaluated by calculating their percentage accuracy, precision, F-1 score, and sensitivity. The work performance was evaluated by measuring the amount of data successfully predicted positively compared to all data positively predicted, including both true and false positives or precision of the models. In this case, we made predictions of the 14 features and whether, after entering the model, these features produced true output in accordance with reality or whether the participant’s SBP or DBP decreased after consuming the medication based on the features included. The sensitivity or recall of these model algorithms was also measured, that is, the number successfully predicted as positive compared to all positive data. The sensitivity illustrates how many models have missed predicting the decreased SBP and DBP of participants who should have been predicted as their blood pressure decreased after taking medicines. The F-1 score was also calculated, that is, the harmonic mean of precision and recall, with the best F-1 score being 1.0 and the worst value being 0. Representationally, a good F-1 score indicates that the classification model has good precision and recall.

### Study Population

The medical records of 100 men with a history of smoking and hypertension were randomly sampled from medical records between July and November 2022 from several hospitals in Yogyakarta, Indonesia. The incidence of systolic and diastolic decline was predicted in these patients using several machine learning models (response variables). The response variable (*y*1) was decreased SBP after taking antihypertensive drugs, *y2* is the decrease in DBP, and the feature predictors (*X*) were smoking-related information, sociodemographic status, hypertension, and several other conditions related to blood pressure (Table 5).

### Data Preparation

The primary data from 100 medical records were filtered, and text variables were transformed into numerical, scaled datasets, and normalized before correlation statistics were performed and visualization heat maps constructed for each feature (Fig. 2). Finally, recursive feature elimination (RFE) was performed to determine the rank of each feature or variable that produced the best prediction model (Table 5).

### Understanding SBP and DBP Before and After Taking Medication

Before further data analysis, the features that influence a decrease in SBP and DBP after taking hypertensive drugs were determined. A *t* test was performed to determine whether there was a difference between SBP and DBP before and after taking medicines.

### Machine Learning Algorithms

To compare machine learning algorithms, the study population was split into a “training” group, in which the features included in algorithms were derived, and a “test” dataset. The “training” dataset was derived from a random sampling of 80% of the extracted data set, and the validation set data comprised the remaining 20%.

### Model Evaluation

The performance of the ML classifiers was compared using accuracy, precision, recall, F-1 score, and sensitivity. The development of the machine learning algorithms in the training and test datasets was completed using Python 3.7 (Python Software Foundation, Wilmington, DE, USA). The ANN was designed using the MLP Classifier (hidden layer size = 3, maximum iterations = 5).

## Results

The participants ranged in age between 25 and 74 years (mean 52 years), and most participants were 55 − 64 years and over 65 years (48%), retired, and had suffered from hypertension for an average of 6.49 years ( Tables 1 and 2).

**Table 1:** Participants’ characteristics

	Age	Frequency	%
Variable	25–34 (early working age)	18	18
	35–44 (middle age)	8	8
	45–54 (pre-retirement age)	21	21
	55–64 (retirement age)	24	24
	> 65 (elderly)	24	24
	Total	100	100
Work	State civil apparatus	34	34
	Private employees	18	18
	Self-employed	6	6
	Indonesian Armed Forces	4	4
	Student	2	2
	Pensioner	36	36
	Total	100	100
Types of cigarettes	Cigarettes	98	98
	E-cigarettes	2	2
	Total	100	100

*The duration from when the participant started smoking to when they quit or sought treatment, measured in years

**The number of cigarettes smoked daily in the past

**Table 2:** Smoking history and blood pressure of respondents

Variable	Mean ± SD	Minimum	Maximum
Age	52.12 ± 12.9	25	74
Smoking time*	10.04 ± 5.5	1	20
Number of cigarettes consumed daily*	9.26 ± 3.3	4	20
Duration of Hypertension	6.49 ± 4.19	1	15
Duration of consumption of doctor’s medications	5.5 ± 4.4	1	15
Blood technology before going to the community health center (puskesmas)/before taking hypertension medicine			
SBP	158.20 ± 10.1	130	180
DBP	98.70 ± 5.7	60	100
Blood pressure after taking doctor’s medication			
Systolic	151.10 ± 12.2	125	170
Diastolic	87.40 ± 4.5	80	95

Before taking any medication, their average systolic pressure was 158 mmHg, and diastolic pressure was 98 mmHg. After taking medication, their blood pressure decreased to 151 mmHg for systolic and 87 mmHg for diastolic pressure (Table 2).

The participants had smoked for an average of 10 years, and most quit after seeking medical treatment. Around 98% smoked cigarettes and 2% smoked e-cigarettes (Table 1), and the average number of cigarettes consumed daily was nine (Table 2).

**Fig. 1:**
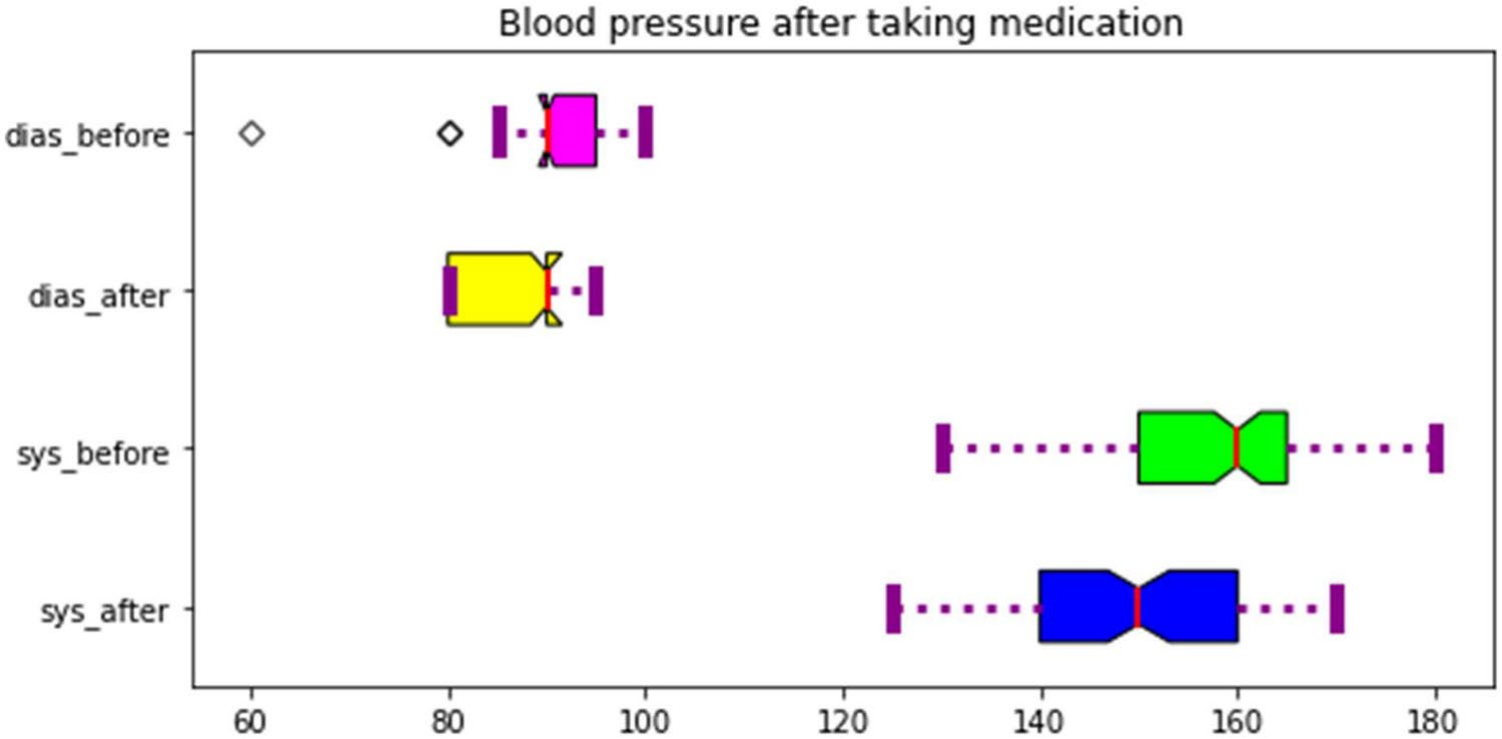
SBP and DBP before and after taking hypertensive drugs

An overview of the differences between SBP and DBP after taking antihypertensive drugs is shown in Fig. 1.

According to the *t* test analysis, there was a significant decrease in both systolic and diastolic pressure after treatment with antihypertensives (Table 3).

**Table 3:** A *t* test analysis of the SBP and DBP before and after given antihypertension drugs

Variable		Before taking medication	After taking medication	Difference	*p*-value^a^
		Mean ± SD			
Hypertension (mmHg)	Systolic	158.20 ± 10.1	151.10 ± 12.2	7.10 ± 8.82	0.000*
	Diastolic	90.70 ± 5.7	87.40 ± 4. 5	3.30 ± 6.7	0.000*

^*^Significant at 0.05 level

^a^Dependent *t* test

The lowest difference in SBP after antihypertensive drug treatment was − 35 mmHg and the maximum was + 10 mmHg with a mean of -7 mmHg. The minimum diastolic pressure change was − 20 and the maximum was + 30 mmHg, with a mean of − 3.3 mmHg. This indicates that even after antihypertensive drug treatment, the patients still experience an increase in SBP and DBP (Table 4).

**Table 4:** Difference between SBP and DBP before and after given antihypertension drug

No.	SBP	DBP
Count	100	100
Mean	− 7	− 3.3
STD	8.8	6.78
Min	− 35	− 20
25%	− 10	− 5
50%	− 10	− 5
75%	0	0
Max	10	30

**Fig. 2:**
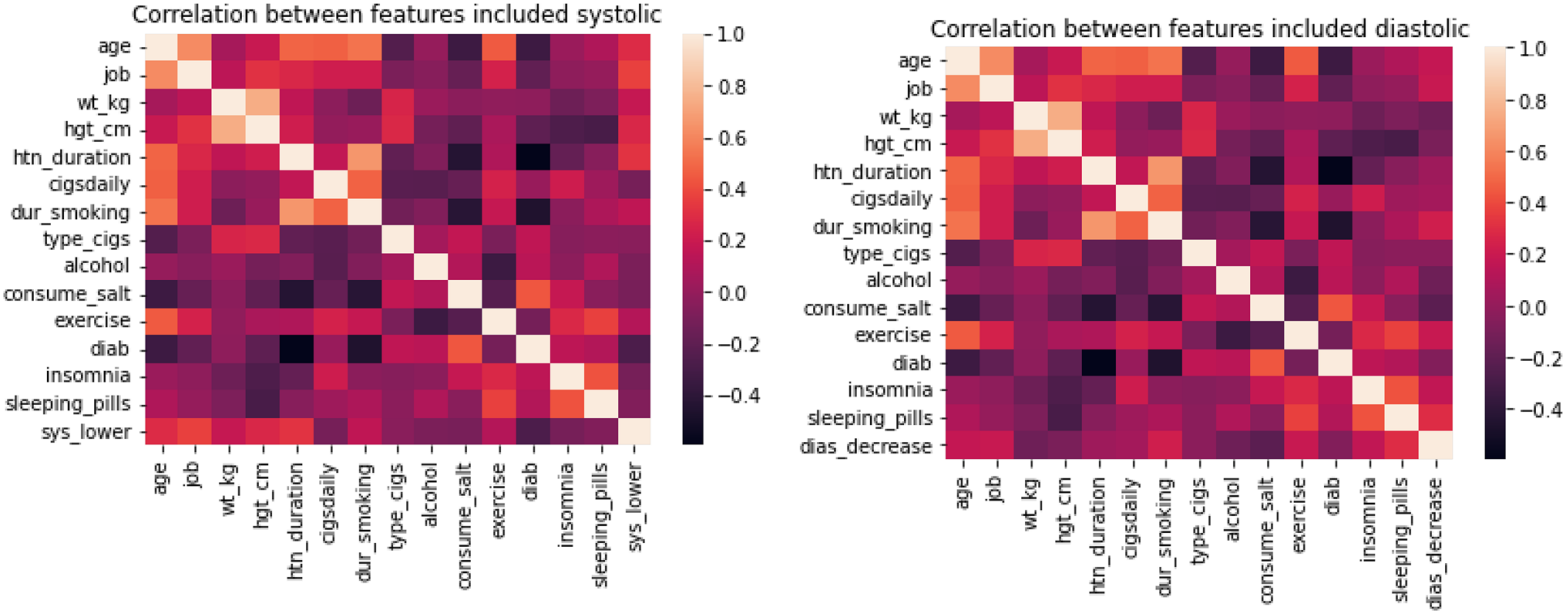
Multilinear regression of decreased SBP and DBP based on features included. Correlation between the decrease of SBP and DBP and the included features. The heatmap provides a graphical representation of the correlation matrix with different variables. High correlation is represented by dark orange

Figure 2 provides an overview of the correlation between the decrease of SBP and DBP after taking antihypertensive drugs and included-excluded features.

Of the features obtained from the 100 randomized medical records, the ten factors most correlated in order with a decreased SBP after taking antihypertensive drugs were age, duration hypertension, occupation, height, diabetes, weight, duration of smoking, exercise, the number of cigarettes smoked per day, and a high salt diet. In comparison, the factors most correlated to the decrease in DBP were age, the duration of smoking, salt consumption, occupation, exercise, the use of sleeping pills, insomnia, weight, consumption of alcohol, and height (Table 5); Table 6 shows output variables (*y*1) for the decrease in SBP and (*y*2) for DBP.

**Table 5:** Input variable (*X*) and order based on the highest correlation to an output variable SBP (*y*1) and DBP (*y*2*)*

No.	Feature used	Feature type	Information	Feature rank selection (most correlated)	
				SBP	DBP
1.	Age	Numeric	Age in years	1	1
2.	Job	Nominal	Job types: 1. Civilian workers 2. Private employees 3. Entrepreneurial 4. Armed forces of the Republic of Indonesia 5. Housewives 6. Student 7. Pensioner 8. Miscellaneous	3	4
3.	Weight	Numeric	Weight (kg)	6	8
4.	Height	Numeric	Height (cm)	4	10
5.	Hypertension duration	Numeric	Duration of patients who have been in the condition (year)	2	14
6.	Cigs daily	Numeric	Number of cigarettes consumed daily	9	12
7.	Duration of smoking	Numeric	How long have patients used to smoked cigarettes (year)	7	2
8.	Type of cigarettes	Nominal	Type of cigarettes consumed: 1. Conventional cigarettes 2. Electric		13
9.	Alcohol	Nominal	1. For consumed 2. For not consumed	12	9
10.	Salty food	Nominal	1. For consumed 2. For not consumed	10	3
11.	Exercise	Nominal	1. For doing exercise 2. 2. For not doing exercise	8	5
12.	Diabetes	Nominal	1. Having diabetes 2. Not having diabetes	5	11
13.	Insomnia	Nominal	1. Having insomnia 2. Not having insomnia	11	7
14.	Sleep pills	Nominal	1. Consumed sleep pills 2. Not consumed sleep pills	13	6

**Table 6:** An output (*y*1 *and y*2) variable

No.	Featured	Feature type	Information
1.	SBP (y1)	Nominal	Systolic blood pressure decreased after taking medicines: 1. Yes, decreased 2. No, not decrease
2.	DBP (y2)	Nominal	Diastolic blood pressure decreased after taking medicines: 1. Yes, decreased 2. No, not decrease

The machine learning performance of NB, DT, ANN, and LoR was good as evidenced by the high precision (≥90%), accuracy (≥84%), and sensitivity (≥80%) for SBP and DBP (Table 7).

**Table 7:** Machine learning performance

No.	Type	Precision (%)	Accuracy (%)	F-1 score (precision and recall)	Sensitivity
1.	Naïve Bayes				
	SBP	96	84	78	93
	DBP	96	85	89	91
2.	Artificial neural network				
	SBP	90	85	87	88
	DBP	90	95	93	94
3.	Logistic regression				
	SBP	90	95	93	94
	DBP	90	95	93	94
4.	Decision tree				
	SBP	94	93	93	80
	DBP	96	93	94	88

## Discussion

The results show that the antihypertensive drugs used in this study do not necessarily reduce SBP and DBP. This is in line with research conducted by Marco et al. investigating the mean decrease in SBP and DBP achieved by antihypertensive drugs which showed that several sociodemographic factors such as sex, ethnicity, and obesity are associated with the antihypertensive response [25].

The machine learning performed well in predicting whether a person with hypertension with certain characteristics would experience a decrease in SBP and DBP after taking antihypertensive drugs. Machine learning can be used for very large datasets and is an effective predictor when used with the features determined as affecting blood pressure. According to the performance indicators of precision, accuracy, precision and recall, and sensitivity, logistic regression has the most stable performance, followed by the decision tree, artificial neural network, and Naïve Bayes. Both the artificial neural network and logistic regression were equally good at predicting a decreased SBP, whereas logistic regression was better at predicting a decreased DBP.

### Strength

This is the first study to investigate the decreased SBP and DBP of male hypertensive patients with a history of smoking history using machine learning models. This is a real condition study as the participants were hypertensive males selected from the public health center (puskesmas) medical records between July and November 2022.

### Limitation

The sample was relatively small, so the machine learning models should be trained and tested using larger datasets and more features to confirm these results and increase the accuracy of the predictions. Not all blood pressure data in this study was measured using enumerators because the participants were prescribed hypertension treatment by their doctors.

## Conclusion

The machine learning models Naïve Bayes, artificial neural network, logistic regression, and decision tree can be used to predict the decrease in SBP and DBP in hypertensive patients who are taking antihypertensive drugs. Since the decrease in SBP and DBP can be influenced by many factors not considered in this study such as patient compliance with taking drugs, obesity, and types of drugs, future research involving larger datasets and more features is needed.

## Data Availability

The data is available upon request.
